# Ergodicity and parameter estimates in auditory neural circuits

**DOI:** 10.1007/s00422-017-0739-5

**Published:** 2017-10-29

**Authors:** Peter G. Toth, Petr Marsalek, Ondrej Pokora

**Affiliations:** 1 Institute of Pathological Physiology, First Medical Faculty, Charles University, U Nemocnice 5, 12853 Prague 2, Czech Republic; 2 Max Planck Institute for the Physics of Complex Systems, Noethnitzer Strasse 38, 01187 Dresden, Germany; 3 Czech Technical University in Prague, Zikova 1903/4, 16636 Prague 6, Czech Republic; 4 Department of Mathematics and Statistics, Faculty of Science, Masaryk University, Kotlarska 2, 61137 Brno, Czech Republic

**Keywords:** Auditory pathway, Ergodic theory, Ergodicity, Circular statistics, Interspike interval, Probability distribution function, Sensory modality, Spike timing jitter, Vector strength

## Abstract

This paper discusses ergodic properties and circular statistical characteristics in neuronal spike trains. Ergodicity means that the average taken over a long time period and over smaller population should equal the average in less time and larger population. The objectives are to show simple examples of design and validation of a neuronal model, where the ergodicity assumption helps find correspondence between variables and parameters. The methods used are analytical and numerical computations, numerical models of phenomenological spiking neurons and neuronal circuits. Results obtained using these methods are the following. They are: a formula to calculate vector strength of neural spike timing dependent on the spike train parameters, description of parameters of spike train variability and model of output spiking density based on assumption of the computation realized by sound localization neural circuit. Theoretical results are illustrated by references to experimental data. Examples of neurons where spike trains have and do not have the ergodic property are then discussed.

## Introduction

The ergodicity concept was introduced among others by Ludwig Eduard Boltzmann (1844–1906; after 1870; Boltzmann^1898^) in statistical physics in his kinetic theory of gases. The ergodic hypothesis states that an average taken over a smaller set of particles, or just one particle, and a longer time period should be equal to the average over a larger set of particles and shorter period of time. Systems fulfilling this hypothesis are called ergodic.

The elementary neural signals are recorded as all-or-none events, action potentials, or spikes, which occur in volleys called spike trains. This is called unit activity. Neurons in most neural nuclei and most brain regions in many species have been recorded. More is known about this in the visual pathway (Maunsell 1992; Van Essen and DeYoe 1995; Marsalek et al. 2017) as compared to the auditory pathway (Syka 2002). The question was always: What stimulus drives a given particular neuron to its highest firing rate (Barlow 1972; Koch 2004)? Relation of the best *modality* to firing rate is denoted as the *rate code* (Koch and Laurent 1999), which implies the highest throughput of firing in neurons. The highest firing rate neurons in humans are in the auditory pathway, and they typically do not exceed a rate of $R=$ 500 per second.

*Modality* is any sensory quality, visual, auditory or other. In a generalized sense in which we use the word here, it can be either an objectively measurable quality, or subjectively quantifiable quality, or even just subjective quality without a quantitative aspect. In a broader sense, it is used also as imaging modality or modality of experimental recording like neuronal firing rate. The concept of modality was introduced in the field of psychophysics by Gustav Theodor Fechner (1801–1887; Fechner 1860; Mountcastle 1957; Kandel et al. 2000).

Another general property of neural signaling is specificity. Most brain regions have a dedicated functionality, which can be seen in sensory pathways. Let us give examples in relation to the five major senses. In vision, specific quadrants of the retina project into corresponding parts of the visual field in the visual pathway, which is called retinotopy. In the auditory pathway neuronal projections are organized according to their characteristic frequencies, which is called tonotopy. In the somatosensory and pain projections the localization on the surface of the body is present up to the projections into the neocortex (Mountcastle 1957). In the olfactory pathway, projections of dedicated lines from olfactory epithelium make up the mosaic of a smell sensation (Marsalek 1994). In the gustatory sense, the spatial organization is analogous to mechanosensory projections (Brozek et al. 1979). All of these topical encodings are called *labeled line* codes and are present in the sensory pathway all the way up to the cerebral cortex. They are the basis of the massive parallelism, which enables fast (motor) reaction times realized by a mass of relatively slow neurons.

Several questions arise: Under what conditions is it meaningful to characterize the assembly of neuronal population by its mean firing rate? What other parameters shall we use? Is it meaningful to average neural firing over given time? Are neural populations homogeneous, so we can average over populations? When can we exchange the population and time average and get the same result, and when can we not do this?

In description of neural population activity, the ergodicity concept can have several possible meanings (Lehky et al. 2005). We use the ergodic property in the sense mentioned above: as interchangeability of averaging over time and neuronal population in spike train neural coding. Let us empirically observe sample statistical characteristics of spike trains. Based on examining the data, the ergodic hypothesis holds when such characteristics taken over longer time with fewer neurons are equal to those in less time with more neurons.

Let us show examples where ergodicity hypothesis holds and does not hold. We propose that ergodicity *holds* in the processing of the low-frequency sound azimuth by the auditory brainstem in man. This is because the unitary computation takes less time during the duration of one sound cycle than the length of any consciously perceived time interval. For example, in sound frequency $f_\mathrm {S} = 100$ Hz this range is $T = f^{-1}_\mathrm {S} = 10$ ms, ten times faster than typical reaction times. Human reaction times range from *circa* 100 to 200 ms (Thorpe et al. 1996). Reaction times depend on the length of the efferent neural connection to muscles involved, with the furthest being a foot on a brake pedal. Auditory stimuli are as powerful as visual in eliciting motor reactions. Strong acoustic stimuli can be tested by startle reflex in laboratory rodents. Rapid serial auditory pipelined stimuli analogous to visual are described in the psychoacoustic literature Vachon and Tremblay (2005).

On the other hand we propose that ergodicity *does not hold* in the processing of complex sounds such as elements of speech. The neural processing over a longer time cannot be replaced by the population ensemble, since the stimulus must be processed as a whole. Many neural codes use labeled lines, like tonotopic or retinotopic organizations in hearing and vision. Most of these organizations we regard as ergodic. One can conjecture about ergodicity based on the time needed to process the entire stimulus leading to the unitary percept. When the duration of this unitary stimulus is short, it is more likely to be ergodic, as opposed to when the unitary duration is longer.

This leads us to a *prospective* application of the ergodic concept. Observation of ergodicity in a particular modality will help uncover how this modality is encoded. We present here an example of parameter estimation in spike trains with a periodic component imposed by sound period. Sanda and Marsalek (2012) have shown that same accuracy of incoming sound azimuth can be obtained by either a longer processing time of fewer neurons, their firing statistically independent and identically distributed, or alternatively, by a shorter processing time and more neurons. Our present study adds further details to this observation.

We use a simple model of auditory spike trains based on fitting of receptive fields of auditory pathway neurons. This can be viewed as a simplification of models by Heinz et al. (2001) and Lopez-Poveda and Meddis (2001). The prospective study presented here is intended as a simple analytical continuation of Sanda and Marsalek (2012), where values of binaural neuron model parameters were obtained using numerical simulations.

The first neuron of the auditory pathway collecting the information from both the left and right side is the neuron of the medial superior olive nucleus. Assuming typical spike trains are entering the MSO, can we calculate the standard deviation of spike timing, or timing jitter from vector strength of spikes in relation to sound phase difference? Based on this, can we estimate parameters of the symmetrical beta density, describing spike timing, from the timing jitter?

Periodicity of sound can range from aperiodic noise to harmonic tones. The sound phase in cochlea can be defined in relation to smoothed instantaneous frequency and at least two points in envelope of incoming sound. Therefore phase might not be defined when noise is introduced by statistical formalism. Low frequency sound localization works in both cases, as in the former the phase reset is due to the sound onset or amplitude modulation. In the former, only the interaural time difference (ITD) is well defined. In the latter case, both the ITD, and the interaural phase difference (IPD), are defined as meaningful signals. In the latter case the probability density functions (PDFs) on the support of the real axis will change into PDFs on the interval $[0, 2\pi ]$, described by circular statistics Mardia (1972). Definitions of these quantities follow. Most of the mathematical formalism here is just elementary calculus and all symbols and numerical constants are duly introduced and explained.

## Definitions, methods, models

### Vector strength

Vector strength measures the periodicity of unit impulses, like neural spikes. This periodicity may relate to stimulus periodicity like, for example, low frequency sound envelopes. Vector strength values range from 0.0 to 1.0. Each unit impulse (spike) is treated as a unit vector with particular phase. Let us start with the definitions of the vector strength Gumbel et al. (1953) and Goldberg and Brown (1969) and of the statistical distributions we use Cipra (1994). Let us have sample phases $\varphi _i$, $i=1, 2, \ldots , N$ relative to phases of a given master periodic function. Pure tone is an example of a sound stimulus that elicits the spikes with these phases. A spike train is a response to this stimulus. Discrete vector strength of the sample $\varphi _1, \ldots , \varphi _N$ is defined as the quadratic mean of $\cos$ and $\sin$ of the phases:

1 $$\begin{aligned} r(\varphi ) = \frac{1}{N} \sqrt{\left( \sum _{i=1}^N \cos \varphi _i \right) ^2 + \left( \sum _{i=1}^N\sin \varphi _i \right) ^2}. \end{aligned}$$

If the phase $\varphi$ space is continuous and can attain all values from an interval of $[0,2\pi )$ with the probability density function $g(\varphi )$, continuous vector strength is defined as

2 $$\begin{aligned} r(\varphi ) = \sqrt{\left( \int _0^{2\pi } g(\varphi ) \cos \varphi \ \mathrm{d}\varphi \right) ^2 + \left( \int _0^{2\pi } g(\varphi ) \sin \varphi \ \mathrm{d}\varphi \right) ^2}. \end{aligned}$$

In terms of the modulus of a complex number, this can be rewritten as

3 $$\begin{aligned} r(\varphi ) = \left| \int _0^{2\pi } g(\varphi ) \exp (i \varphi )\ \mathrm{d}\varphi \right| , \end{aligned}$$

where $\exp$ denotes the exponential function and *i* stands for the imaginary unit. The three definitions (1), (2), (3) are equivalent and yield $r\in [0,1]$. Further details can be found in (van Hemmen 2013). Alternatively, if the synchronization is described by time intervals $t_i \in [0,T]$ between the specific point of the given master periodic function and the response spike, previous definitions of the vector strength can be expressed in terms of

4 $$\begin{aligned} \varphi _i = \frac{2 \pi t_i}{T} = {2 \pi t_if}, \end{aligned}$$

where *T* is the period of the master function (i.e. period of the sound). In the case of sound, we alternatively use fundamental sound frequency $f = 1/T$ to get rid of the compound fractions, where it is appropriate.

### Beta probability density function

In order to scale the spike times distribution by a scale coefficient *s*, we introduce a three parameter version of the standard beta distribution. This way we can use appropriate forms of the distribution regardless of whether the support is on the interval [0, 1), or on the interval $[0, 2\pi )$. This can be decided by setting values $s=(2\pi )^{-1}$ or $s=1$. Continuous values of $s\in (\delta ,1]$ with small $\delta$ describe a continuously parameterized phase locking of input spikes. We need the scaling parameter for relation of the spike train instances to the sound phase, as it will be shown in the beginning of Results. The beta distribution has then a probability density function written as:

5 $$\begin{aligned} f_\mathrm{B}(t,a,b,s)= & {} sB(a,b)^{-1}(t/s)^{a-1}(1-t/s)^{b-1}\nonumber \\&\mathrm {H}(t/s)\mathrm {H}(1-t/s), \end{aligned}$$

with variable *t* and parameters $a, b \ge 1, s > 0$. The formula of the distribution is normalized by Euler beta function *B*(*a*, *b*) to give unity integral (Cipra 1994). Its range is cut off by Heaviside function $\mathrm {H}(t)$, $\mathrm {H}(t)=1$ for $t>0$, otherwise $\mathrm {H}(t)=0$. The mean $\mu$, standard deviation $\sigma$ and coefficient of variation $C_\mathrm {V}$, of the beta distribution expressed in parameters *a*, *b* and *s* are:

6 $$\begin{aligned} \mu= & {} \frac{sa}{a+b}, \sigma = \sqrt{\frac{s^2ab}{(a+b)^2(a+b+1)}}, C_\mathrm {V} = \frac{\sigma }{|\mu |} \nonumber \\= & {} \sqrt{\frac{b}{a(a+b+1)}}. \end{aligned}$$

Numerical computations with descriptive circular statistics (Mardia 1972) are available as a library package in Matlab (Berens 2009).

### Auditory model

We use the model by Sanda and Marsalek (2012). The model uses simplified realization of auditory periphery. The auditory periphery starts in the inner ear and ends at the auditory thalamus, called the corpus geniculatum mediale. The spike trains originate in the cochlea. The cochlea is an active mechanical-to-electrical transducer. An analogy to analog-to-digital conversion is used to describe cochlea. An analog electrical signal, which can be captured as a cochlear microphonic signal, is converted into a digital signal of spike trains. The rest is processed as neural computation. The auditory periphery diverts spike trains into two branches. The principal branch processes sound content. The branch of the localization part of the auditory pathway depends on comparison of inputs from the left and right ear, which is realized in a third neuron, counted from the cochlea, belonging to the medial or lateral oliva superior. These two branches then constitute sound object and sound localization in the central part of the auditory pathway.

The model describes in more detail the localization branch of the neural circuitry, circuit of medial oliva superior. Sound signal is represented by spike trains produced by phenomenological neuronal units, which describe first neuron in auditory nerve. The second neuron is in the cochlear nucleus. All the diversity and specializations of these neurons are neglected. The model contains diverging outputs of the cochlear nucleus into the object and localization branches.

The parts of cochlear processing and first neuron of the model are similar to Heinz et al. (2001). When the phenomenological peripheral part in it was replaced by more detailed cochlear mechanisms taken from Lopez-Poveda and Meddis (2001), the major difference between the two models was that the more detailed contained more low pass filtering properties. Important features of any model discussed above are nonlinear processing of sound intensity, tonotopic organization, sound phase propagation through the pathway and representation of all auditory modalities in spike trains. The neuronal representation of these modalities in spike trains can be intrinsic and extrinsic, DeWeese et al. (2003) and Lehky et al. (2013). Extrinsic encodings are typically faster, they can be multiplexed in small chunks and, therefore, may have the ergodic property in the sense discussed here.

Even in the absence of sound, most auditory neurons exhibit spontaneous activity. In the model we neglect this activity. In construction of the model we can simplify the threshold definition. Threshold level for pure tones is dependent on sound frequency.

**Fig. 1:**
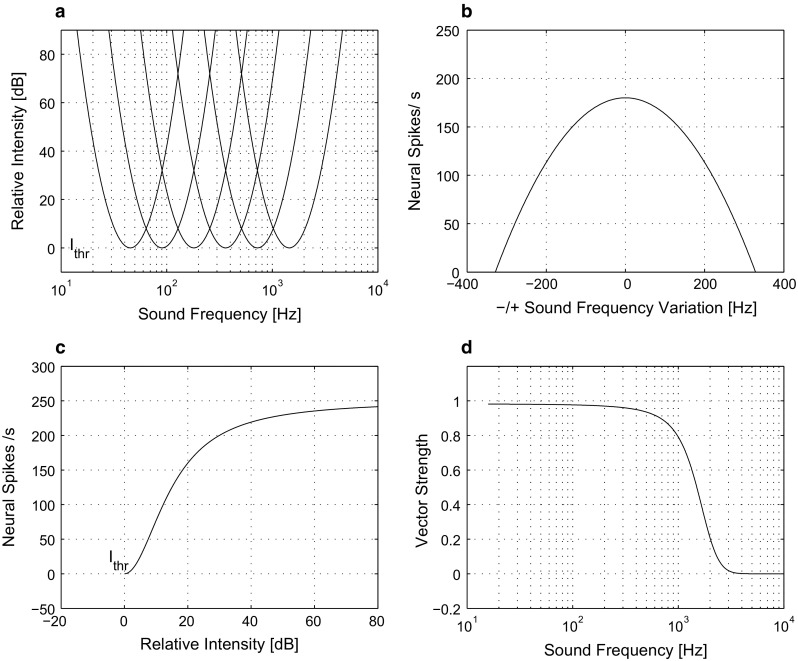
Auditory neuron firing in response to sound. **a** Regions of responses of individual neurons in the spiral ganglion, which is the originating site of the auditory nerve. The *x*-axis is in logarithms of sound frequency and the *y*-axis is in decibels, which are logarithmic units of sound intensity. The idealized response regions have the shape of parabolas and the threshold $I_\mathrm {thr}$ is here schematically assumed to follow the line of $y = 0$. The vertices of these parabolas are at the point of characteristic frequency of individual neurons ($f_{\mathrm C}$, 0). **b** The firing bandwidth, measured in Hz of sound frequency, for sound frequencies adjacent to the best frequency. The *x*-axis in this panel is on a linear scale. **c** The panel shows how neuronal firing is saturated. The *y*-axis shows the firing rate recruitment in response to rising sound intensity, and the *x*-axis shows decibels, starting from the threshold $I_\mathrm {thr}$ of individual neurons. This is a prototypical neural response without spontaneous activity. **d** The monotonic sigmoidal decrease of the vector strength with increase in sound frequency, which is observed in the majority of all the neurons in the auditory pathway

Individual parabolic tuning curves are shown in Fig. 1a on a logarithmic scale on the *x*-axis. They have vertices at given characteristic frequencies (CFs). Threshold intensity is denoted $I_\mathrm {thr}$ on this fitted curve. This can be compared to data of Joris et al. (1994a, b, 2006a). The example of discretization to *N* tuning curves with index *i*, $i \in \{1, 2, \ldots , N=20\}$ is not arbitrary, as all three major manufacturers of cochlear implants use $N\approx 20$ stimulating electrodes (Drapal and Marsalek 2010). Examples of six tuning curves are shown in Fig. 1a. With proportionality parameter $K = 32$, the receptive fields are: $N_i(x) = K(x-f_\mathrm {CF}(i))^2 + y_\mathrm {CF}(f_\mathrm {CF}(i))$, where *x* is the frequency variable on the *x*-axis and *y* is the parabolic threshold function on the *y*-axis. We must transform sound frequency $f_\mathrm {S}$ into logarithmic units. $f_\mathrm {CF}(i)$ is a set of representative characteristic frequencies. Figure 1b shows responses of model neurons to neighboring frequencies. The dependence is a concave parabolic function with its maximum at the CF and tails descending towards the side frequencies. On the *y*-axis is a linear firing rate.

Even though this is a simplified and canonical model of auditory periphery, when it was used as input to the MSO model circuit, its activity was comparable to a more complicated series of Meddis models, which can be followed from Lopez-Poveda and Meddis (2001). The rise of the response rate with rising intensity is given by a sigmoidal function. The sigmoidal shape comes from the molecular mechanism of ion channel opening in hair cells following the kinetics of Michaelis and Menten (1913), Naka and Rushton (1966), Sclar et al. (1990) and Camalet et al. (2000),

7 $$\begin{aligned} R(I) = R_{\max }\frac{I^n}{I^n +I_{50}^n}, \end{aligned}$$

where $I$ is the relative input intensity, $I_{50}$ is the intensity evoking 50% of maximal response $R_{\max }$ and *n* is the exponent determining the steepness of this saturation function. For the purpose of this study, we set $R_{\max }= 250$ s$^{-1}$ and $n = 2$. Sound intensity $I$ ranges from hearing threshold, $I_\mathrm {thr} = 0$ dB, to pain threshold, which is $I_{\max }\approx 130$ dB, for the reference sound frequency (1 kHz). We refer to these values only as to relative intensity decibels. The function $R(I)$ is called the rate-level (in case of sound, where term level is used), or the rate-intensity function (in other modalities). Vector strength decreases with increasing sound frequency, as described in Marsalek (2001),

8 $$\begin{aligned} r(f_\mathrm {S}) = \frac{1}{1 + \exp (f_\mathrm {D})}. \end{aligned}$$

The frequency difference $f_\mathrm {D}=(f_{\max }-f_\mathrm {S})/f_{\max }$ enters this empirical formula for $r$, with $f_{\max }=~1$ kHz. This equation as well as (7) are saturating curves with maxima $R_{\max }$ and $f_{\max }$.

### Ergodicity

All the results presented in this paper are calculated under the ergodic assumption. While many theoretical and experimental results are obtained under ergodic assumption, the situations where ergodicity is violated would yield higher than expected variability. This in turn would lead to experimental designs, which are difficult, or even impossible to reproduce. This non-reproducibility would produce a negative result and these as such are more difficult to find throughout the literature. In this study, we first mention designs where ergodicity holds and later describe designs correctly pointing to situations where ergodicity is not fulfilled.

The ergodic assumption is used in the method of recording various sensory evoked potentials, where the spatially averaged gross potential signal is composed of individual neuronal contributions. In Results we present dependencies of vector strength and variation of the interspike interval on parameters of neuronal models. In general, descriptions of spike trains as point processes without memory use the ergodic assumption.

Ergodicity is not present in the binary coding model by DeWeese et al. (2003). The binary coding model is proposed as a contrasting mechanism to the rate coding model with the ergodic property. These authors use the binary coding model as an explanation for sparse spiking in the recordings from rat auditory cortex. Another theoretical and experimental demonstration of higher neuronal firing variability is in Cecchi et al. (2000). These authors analyze the response from the cat visual cortex.

## Results

### Vector strength of neural firing

Vector strength *r* of a spike train with the beta distribution (5) of ISIs can be parameterized by *a*, *b* and scaled by *s*. For the value of $s = 1$ the distribution has nonzero image for $t\in (0,1)$. The lowest natural number non-trivial parameters of the density are $a = b = 2$. The sound phase defined on the interval $[0,2\pi )$ must be normalized to [0, 1) by setting $s = 1/2\pi$. Assume an example spike train, where the spike phases relative to sound phase are distributed according to beta density (5). For this beta density the scaling parameter *s* can attain values from $0 \le s < 2\pi$ and $r$ can be expressed as

9 $$\begin{aligned} r= 12s^{-3}|s\cos (s/2)-2\sin (s/2)|. \end{aligned}$$

For any given natural *a* and *b*, $r$ can be expressed with the use of hyper-geometric function. However, a formula for all arbitrary values of *a* and *b* does not exist. From (6) we immediately see that the mean of $f_\mathrm{B}$ for $a=b$ is $\mu (f_\mathrm{B}(t,a,a,1))=0.5$. With $s=1$ the value of parameters $a=b=4$ appears a better parameter choice for spike trains, since the density tails at points 0 and 1 are smoother, compared to $a=b=2$.

Now the question arises what error we obtain when we replace a spike train histogram resembling a sine function by $f_\mathrm{B}(t,4,4,1)$. The sine function $f_{\sin }$ on support [0, 1] with the same maximum equal to $\max f_\mathrm{B}(t,4,4,1) = 2.2$ is

$$\begin{aligned} f_{\sin } = 1.1(1 + \cos 2\pi 1.1(t - 0.5)). \end{aligned}$$

Its integral $\int _0^1$ equals to 1 after normalization by scaling parameter $s \approx 2.2/2 = 1.1$, as it is required to be a probability density function. We can translate this function such that its peak in interval [0, 1] is aligned with the peak of the given beta density. Its nonzero tails therefore do not end at points 0 and 1 and the pieces of the sine function on the remainder parts of the interval [0, 1], namely [0, 0.0555] and [0.9445, 1], because $0.5 - 0.5/1.1 = 0.0555$ and $0.5 + 0.5/1.1 = 0.9445$, have to be set to 0 by definition (Marsalek and Lansky 2005). A better alternative, non-translated sine function can be obtained by a solution of the nonlinear function for unknown parameter *a*, such that $\max f_\mathrm{B}(t,a,a,1) = 2$. This way we obtain numerically a value of $a = 3.3818$.

**Fig. 2:**
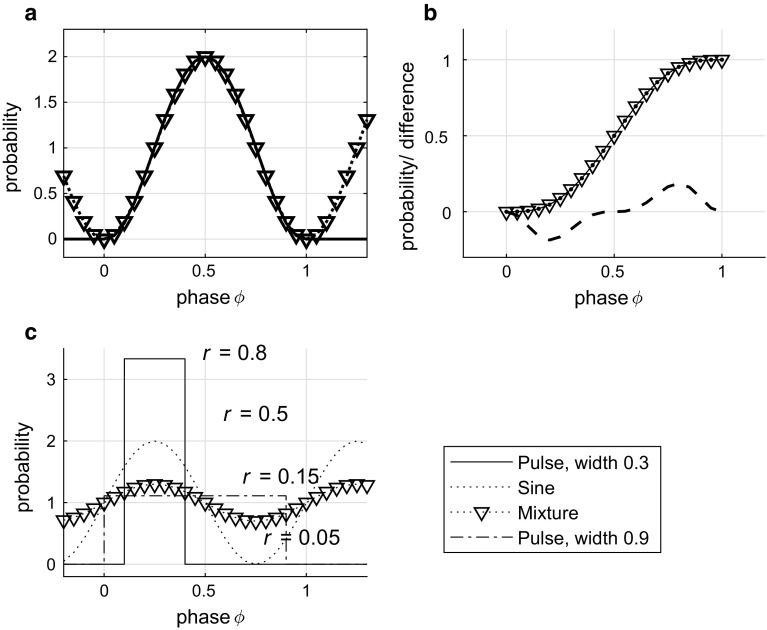
Comparison of circular probability density functions of sine and beta density. **a** Beta density $f_\mathrm{B}$ with parameters $a = b = 3.3818$. The solid line of this beta density closely matches the sine function $f_\mathrm{sin} = 1-\cos 2\pi \varphi$, which is shown by the dotted line with triangles pointing down. Since the sine function is parameter free, another parameter *s* is introduced. **b** The two closely overlapped solid lines show two CDFs of $f_\mathrm{B}(\varphi , 3.38, 3.38, 1)$, dots, and of the $f_{\sin }$ function, triangles. The thick, interrupted line shows the difference between the two CDFs multiplied by 100 to visualize the comparison of the CDFs of the two distributions. **c** This shows how different mixtures of the uniform and sinusoidal distributions attain different values of vector strength. A narrow pulse rectangle has $r=0.8$, a sine function $f_{\sin }$ has $r=0.5$, the curve of triangles shows an arbitrary mixture function with $r=0.15$, and rectangle close to a constant function has $r=0.05$. Details of other parameters are in the text.

Figure 2 shows probability distributions and some of the parameters studied in this section. Panel a shows imposing periodic boundary to interval [0, 1). Panel b shows less than 1% difference of two example CDFs. Panel c shows examples of circular densities and their vector strengths. The narrow rectangular pulse of width 0.3 and height 3.3 has $r=$ 0.8, the sine function with amplitude 2 and period identical to boundary has $r=$ 0.5, the arbitrary mixture function with $1-u =$ 0.7 of rectangle and $u =$ 0.3 of sine has $r=$ 0.15, and the rectangular pulse close to constant of width 0.9 and height 1.11. has $r=$ 0.05.

Figure 2b shows how close the two example densities are. When we used two different tests for normality, the Kolmogorov-Smirnov test, (Smirnov 1948) and the test of Jarque and Bera (1980) implemented in Matlab, neither of the tests showed any significant difference between these two densities in comparison to normal density. (Normal density is not shown on this figure, its graphical comparison to beta can be found in Drapal and Marsalek (2010)). The two densities passed the test as normal density, which is not correct. Let us discuss first the case of the PDF support from minus infinity to plus infinity.

The normal and beta densities clearly differ, as the former has nonzero tails and the latter has zero value tails on support from $-\infty$ to 0 and from 1 to $+\infty$. Over and above, for the periodic support, the corresponding two circular distributions (circular normal, also known as von Mises and sine) also have different analytical properties. The differences in the values they attain are small. Hitherto, analytical formulas of the two distributions are distinct as well. Differentiability is one property analogous to the range $(-\infty ,+\infty )$ functions: the analytic CDF expression does not exist for the circular normal distribution, but does exist for the sine distribution.

In this subsection we have found corresponding parameters of pairs of PDFs on the support of $(-\infty ,+\infty )$ and on the periodic support in the interval [0, 1]. Data recorded in experiment or used in neuronal models must be classified according to their underlying PDFs. Negative results are presented in this subsection on purpose. Of course, once we are aware of the underlying probabilistic nature of data, we can correctly compute parameters and use correct statistical tests. The rather abstract assumption on the probabilistic nature of the PDFs is, therefore, *conditio sine qua non*. Hence, if this essential condition is not followed, one can easily apply inappropriate statistical methods with no warning triggered by the software as the nature of the PDFs has to be determined before the data analysis.

In the following paragraphs we will address the inversion problem by interchanging the dependent and independent variables in Eq. (9) showing dependency of the vector strength $r$ on the parameter *s*. Note that following this equation, we seek relations between parameters (like *d*, *u* and others), standard deviation (spike timing jitter) $\sigma$, variance $\sigma ^2$, and coefficient of variation $C_\mathrm {V}$, of distributions describing spike timing. Given cases where this is not possible analytically, we proceed numerically.

### Variation of the interspike intervals

Let us assume a three-parameter probability distribution of the phase, $\varphi$, on the unit circle given by its probability density

10 $$\begin{aligned} f(\varphi , \alpha , \beta , u)= & {} \frac{1}{\beta - \alpha } \left[ 1 - u\cos \left( 2 \pi \frac{\varphi - \alpha }{\beta - \alpha } \right) \right] \nonumber \\&\mathrm {H}(\varphi - \alpha ) \mathrm {H}(\beta - \varphi ) . \end{aligned}$$

This formula gives a mixture of uniform and sinusoidal distributions, and the parameter $u$ is the weight of the sinusoidal part. Parameters $\alpha$ and $\beta$ are bounds of the support of the distribution. Of course, they must satisfy $0 \le \alpha \le \beta \le 2 \pi$. The weight parameter, $u$, is limited to interval [0, 1]. When $u= 0$, $f(\varphi )$ becomes the probability density of the uniform distribution on interval $[\alpha , \beta ]$. A pure sinusoidal distribution is achieved for $u= 1$. The following subscript *f* denotes phase representation. The distribution is symmetric with mean equal to

11 $$\begin{aligned} \mu _{f}= \frac{1}{2} (\alpha + \beta ) \end{aligned}$$

and variance equal to

12 $$\begin{aligned} \sigma _{f}^2 = \frac{(\beta - \alpha )^2}{12 \pi ^2} \left( \pi ^2 - 6 u\right) . \end{aligned}$$

It can be seen that the variance depends on the difference $\beta - \alpha$ only, not on the actual values of $\alpha$ and $\beta$. Denoting $\delta = \beta - \alpha$ as the length of the support, we can write

13 $$\begin{aligned} \sigma _{f}^2 = \frac{\delta ^2}{12 \pi ^2} \left( \pi ^2 - 6 u\right) , \end{aligned}$$

where $\delta$ can vary between 0 and $2\pi$. Vector strength of this distribution is computed in accordance with (2) or (3). Both give the resulting expression of the vector strength,

14 $$\begin{aligned} r_{f} = \frac{8 \pi ^2 + 2 \delta ^2 (u- 1)}{\delta (4 \pi ^2 - \delta ^2)} \sin \frac{\delta }{2}, \end{aligned}$$

dependent on the length of the support, $\delta$, and the weight, $u$.

The subscript *g* denotes time representation. Let us turn to the temporal representation of the delay, expressed in terms of $t = \varphi T/{2 \pi }$, where *T* denotes the period of the sound. The period is equal to the inverse value of the frequency of the sound, $T = 1 / f_\mathrm{S}$. The transformed probability density *g*(*t*) is calculated in accordance with the integral transform theorem, and it is equal to

15 $$\begin{aligned} g(t, a, b, u)= & {} \frac{1}{b - a} \left[ 1 - u\cos \left( 2 \pi \frac{t - a}{b - a} \right) \right] \nonumber \\&\mathrm {H}(t - a) \mathrm {H}(b - t) , \end{aligned}$$

where the interval [*a*, *b*] is its support, $0 \le a \le b \le T$. The new bounds – parameters are transformations of the original ones, $a =({T}/{2 \pi })\alpha$, $b =({T}/{2 \pi })\beta$. The length of the new support we denote analogously by $d = b - a$. In the new parameterization, the delay distribution has mean value equal to

16 $$\begin{aligned} \mu _{g} = \frac{1}{2} (a + b) \end{aligned}$$

and variance equal to

17 $$\begin{aligned} \sigma _{g} ^2 = \frac{d^2}{12 \pi ^2} \left( \pi ^2 - 6 u\right) . \end{aligned}$$

For calculation of the vector strength, the adjusted variant of (3) is used,

18 $$\begin{aligned} r_{g} = \left| \int _{0}^{2 \pi } g(t) \exp ({2 \pi i t / T}) \mathrm{d}t \right| . \end{aligned}$$

The evaluation of the integral gives an analytical expression, dependent on the parameters *d* and $u$,

19 $$\begin{aligned} r_{g} = \frac{T^2 + d^2 (u- 1)}{\pi d (T^2 - d^2)} T \sin \frac{\pi d}{T} . \end{aligned}$$

**Fig. 3:**
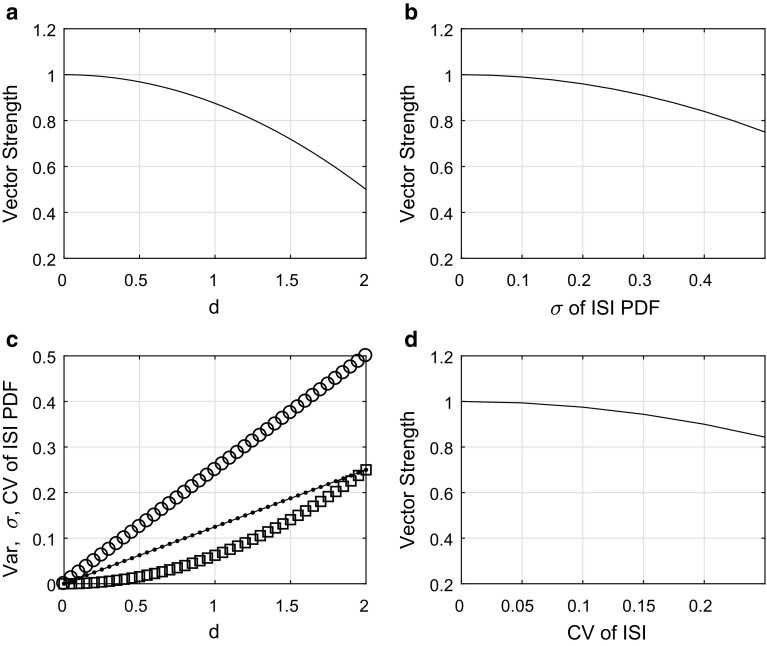
Relation of the vector strength to the jitter and other measures. **a** The dependence of the vector strength $r$ on the width *d* of the interval support. In the limit of the Dirac delta, when $d\rightarrow 0$, $r\rightarrow 1$. **b** The relation of the vector strength $r$, *y*-axis, to the timing jitter $\sigma$, *x*-axis. Because this relation is monotonic, it is invertible within the appropriate definition ranges. **c** Dispersion measures: standard deviation $\sigma$, circles, variance $\sigma ^2$, squares, and the variation coefficient $C_\mathrm {V}$, dots, are shown here as functions of the width *d*. **d** The relation of the vector strength $r$, *y*-axis to the variation coefficient $C_\mathrm {V}$, *x*-axis.

Figure 3a shows this dependence (19) and the other panels b, c, and d show relations between other parameters.

### Model of output spiking density

In a previous subsection we have calculated statistical characteristics of spike trains described by phase locking and spike timing with the use of the compound uniform and sine distribution. Spike timing jitter has probability distribution described by density (15). We assume that the resulting spikes form a renewal process. That means all the interspike intervals (ISIs) have the same probability distribution, and all are mutually independent. In such cases, the stochastic behavior of the spike train is fully described by the distribution of the ISIs.

The following are probabilistic computations with individual spikes, which have recently been referred to as *neuronal arithmetic*Bures (2012) and Bures and Marsalek (2013). Let us fix the time $t = 0$ to a specific point on the sound wave and let us introduce the following notation. The first random variable, *X*, gives the random delay of the time occurrence of a spike; this is given by (15). The second random variable, *Y*, denotes the time occurrence of the subsequent spike: this spike comes at time *T* during the next sound wave with random delay given by density (15). In order to calculate the distribution of the ISIs, it is sufficient to express the random length of the ISI as a difference $Z = (T + Y) - X$ of the two random variables, $T + Y$ and *X*. By the statistical calculus, the probability density function *h* of random variable *Z* is given by convolution

20 $$\begin{aligned} h(t) = g (t - T, a - T, b - T, u) *g (-t, a, b, u) \end{aligned}$$

of probability densities (15) of random variables $(T + Y)$ and *X*. The parameters of function *g* are as before. The first one is shifted by the period *T*, the second is reversed in *t*. These *arithmetic* expressions follow the connectivity in the MSO. In the calculation we use the property of the cosine function that it is an even function, $\cos (-t) = \cos (t)$, and we set $g (-t, a, b, u) = g (t, a, b, u)$.

Ergodicity is used in this calculation in the formulation of the assumption that both inputs are realized by one spike with given PDF in time. As in Drapal and Marsalek (2010), we calculate the resulting convolutions of both sine and beta densities using the symbolic calculation library of the Matlab package. In particular we use the property of the Laplace transform to change the convolution operation into multiplication. The probability density function of the ISIs is then equal to

21 $$\begin{aligned} h(t)= & {} \frac{d - |t - T|}{d^2} + \frac{u^2 (d - |t - T|)}{2 d^2} \cos \frac{2 \pi |t - T|}{d} \nonumber \\&+ \frac{u(4 - u)}{4 \pi d} \sin \frac{2 \pi |t - T|}{d} \end{aligned}$$

for $|t - T| \le d$. This probability distribution has support $[T-d, T+d]$ and depends on the parameter *d* and the weight, $u$. The resulting probability density is left-to-right symmetric. In other words, it has zero skewness. We denote its parameters with the subscript $\mathrm {h}$. Its mean value is equal to

22 $$\begin{aligned} \mu _{h} = T \end{aligned}$$

and variance equal to

23 $$\begin{aligned} \sigma _{h}^2 = \frac{d^2}{6 \pi ^2} (\pi ^2 - 6 u) . \end{aligned}$$

The coefficient of variation is often used for the description of the firing characteristics of a spike train. The derived distribution of ISI has coefficient of variation equal to

24 $$\begin{aligned} {C_{\mathrm {V}h}} = \frac{d}{\pi T} \sqrt{\frac{\pi ^2 - 6 u}{6}} . \end{aligned}$$

Expressing the weight, $u$, from (24) and substituting it into (19), we obtain the vector strength of the output spike train as a function of the coefficient of variation of the ISIs of the input spike train, support interval length *d* and onset time *T*,

25 $$\begin{aligned} r_{h}= & {} \frac{T}{6 \pi d (T^2 - d^2)} \left[ \pi ^2 (d^2 - 6 T^2 C_{\mathrm {V}h}^2) + 6 (T^2 - d^2) \right] \nonumber \\&\sin \frac{\pi d}{T} , \end{aligned}$$

with the parameter *d* controlling the length of both the supports of the delay and ISIs.

**Fig. 4:**
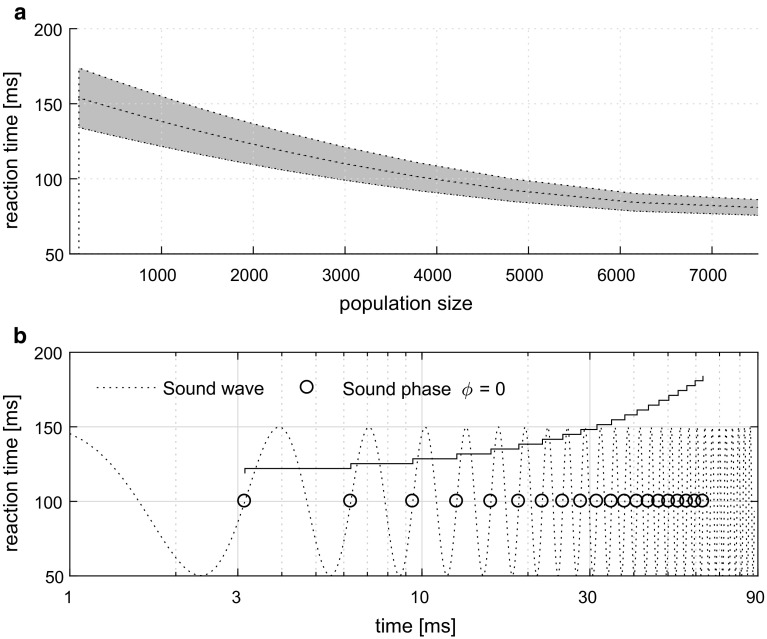
Ergodicity of reaction time related to population size and computation time. Human reaction times are in the vicinity of the following points. On both *y*-axes the reaction times are around 150 ms. **a** Population averaging, with gray bands that illustrate the response variation. **b** Time averaging, which shows the elementary time step increments of successive sound cycles for a range of low sound frequencies. These range from $f_\mathrm {S}\approx$ 200–400 Hz, with a period $T = 1/f_\mathrm {S}$ of 25 to 50 ms. The panel shows an example sound wave of constant frequency $f_\mathrm {S}=$ 318.31 Hz on a logarithmic scale

In this subsection we have presented outputs of the MSO nucleus. The outputs are then transmitted to upper neuronal relays. We assume that during this transmission, spike timing jitter is at least two orders of magnitude smaller than the shortest sound period in question, $\sigma<< T$ and $\sigma<< 1$ ms, Laback and Majdak (2008). A number of lines run in parallel, and as their number increases earlier the ideal observer module signals its output. We call this the reaction time of the model. As the methods used above are generally statistical, we did not write physical units here at all places where they could be added. The results obtained here are applicable in any sensory pathway, where the reaction time is measured. The concept of *neuronal arithmetic* with spike trains is a principle unifying spike train description making them usable in all sensory pathways.

### Ergodicity and reaction time

After obtaining the output of the MSO, we ask: How is the output signaled further up in the auditory pathway? The assumption that all the neurons in this nucleus are responding identically is wrong. In that case there would be no advantage in having more neurons than one. This is probably why parameter variation and stochasticity are beneficial for neural computation and coding.

Spike timing jitter helps in speeding up the reaction time in all sensory modalities. Based on experimental paradigm, the longest reactions originated in the MSO can range from 0.5 s up to 5 s. This would last too long. While detection of auditory stimulus of 50 ms duration is erroneous, typical discrimination azimuth integration time ranges from 150 to 300 ms. The localization branch of the auditory pathway is highly parallel and contains more than few hundred neurons. When the ergodicity is assumed, the reaction time can be divided between jittered responses of individual neurons, Laback and Majdak (2008). The time thus obtained corresponds to the theoretical minimum time required by human listeners to determine the ITD, when the entire brainstem circuit is working in parallel and cooperating on the ITD estimation.

Figure 4 shows a simplified schematic example to illustrate the ergodic assumption in this case. Figure 4a, showing the neural population effect, is the output of *N* copies of one spike neuronal signal propagating from the MSO to the ideal observer module, where the latencies of the spike differ from neuron to neuron due to random variation of spike timing (spike timing jitter). These are results of repeated numerical simulations of a single neuron by Sanda and Marsalek (2012), extrapolated for a population size of between $N=5000$ and 8000 neurons, which is a typical size of the human MSO Philip X. Joris, personal communication.

Figure 4b, illustrates results described in detail in Marsalek and Lansky (2005). In Fig. 4 ibidem was used constant sound frequency of 750 Hz, here we use constant frequency of $f_\mathrm {S} = 318.31$ Hz. While on the previous panel a is shown the population summation, on the panel b is shown repeated neuronal arithmetical “and” operation, which is clocked by sound phase. This can be understood as the time summation effect in individual neuron in generalized sense (since the term neural time summation is usually reserved for the signal integration by individual neuron). The staircase reaction time curve is based on an exact analytical calculation of coincidence detection probability in the coincidence model of the MSO in the paper referred to above. The figure indicates that reaction time is decreasing as a function of population size, but increasing as a function of time. In Fig. 4a the reaction time is diminishing when population is larger. In Fig. 4b the reaction time is growing, as it can be counted by periods of the principal sound frequency. In this subsection we have shown how output of the sensory pathway can be traced by the reaction time.

## Discussion and prospects

### Spike train statistics

In our computations we sought a simple description of spike trains following sound phase. The candidate functions we tried were circular normal and circular beta distribution functions. These are circular counterparts of normal and beta PDFs. A sine is circular and is parameter-free, and it is the simplest of all the circular PDFs. When we use compound density, a weighted sum of uniform and of sine density, we arrive to an arbitrary value of vector strength. We can invert monotonous dependencies of vector strength on standard deviation and other parameters of circular densities. By using this procedure, we can fit the ratio of uniform and sine component to vector strength of experimental spike trains. The last section of results describes how spike density changes when two spike trains from two input pathways undergo neuronal arithmetic operation. As an example operation we use the difference of spike times calculated in the medial superior olive. We calculate the output spike density dependent on the time delay between the right and left side. Finally, we illustrate the neural computation outputs by description of reaction time changes, dependent on population size and on computation time counted in sound time periods.

How are our results relevant for the ergodic hypothesis? Under the ergodic assumption, the individual neurons of neuronal population are assumed to be independent computational units performing neuronal arithmetic operations. Arrival time to outputs of these operations can be used as prediction of reaction times. In this first part of the Discussion we have summarized the results of our computations. In the rest we discuss the Prospects in the field of neural coding in auditory system, particularly in sound localization.

### Comparing experiments and theory

Let us return to the notion of *rate* codes and *labeled line* codes from the Introduction. The idea to stimulate experimentally the neuron of interest to the highest possible firing rate response seems simple. If only all descriptions of neural coding were that simple. Then one could simply say that all the neuron encodes is the mean firing rate. One can imagine this rate is heard by an attentive experimentalist listening to a neuron over an amplifier and loudspeakers. Her peripheral auditory system then encodes the sound pitch of this heard rate again by a *rate* code, which together with the *labeled lines* of her very own auditory nerve generates the impression of the low frequency sound she perceived.

Or if it were only *spike timing* coding in the proverbial grandmother cell, a term coined by Barlow (1972), all of neural coding could be understood in simple terms. However, that is not the case. In the brainstem neural circuit, which we used as an example, it is also important when the neuron of the MSO fires relative to the sound phase *difference* between the two ears.

The *spike timing* needed for the sound azimuth encoding has more than ten times better timing accuracy than is observed elsewhere in the mammalian neural system and its behavioral performance can be even further improved by training.

Goldberg and Brown (1969) were among the first to use real-time online contemporary computers to process the data from the auditory brainstem with time precision down to microseconds. They accomplished a technically demanding experiment, while processing data both on- and off-line. These authors popularized vector strength as a measure of phase locking, or coherence in the neuroscience community. Their experiment was at the beginning of the electro-physiological inquiry of how sound azimuth is calculated in the nervous system. These inquiries and debates continue to the present time.

One of the leading theories of brainstem circuit computation was proposed by Jeffress (1948). This study hypothesized that spike timing relative to the sound phase difference is converted into the labeled line code by a set of delay lines. The axonal, or alternatively dendritic delays compensate for the delay differences between the left and right ears due to the distance the sound must travel in the air. This mechanism was experimentally demonstrated in barn owls, hens and other birds. Let us only mention one example by Fischer and Peña (2009), firstly, because their paper combines experimental data with theoretical concepts, and secondly, because it discusses the other alternative theories of how sound direction is computed.

Without aiming to give a fully comprehensive list, we detail three alternative theories of neural azimuth computation and one example of a purely mechanical solution to the problem. We list them in historical order.

Mammals with small heads, such as rodents, and humans probably employ different mechanisms of azimuth computation than the *(1) Jeffress delay line*, (Jeffress 1948; Joris et al. 1998).

Another theory considers the role of the *(2) cochlear delay* (Schroeder 1977). Phase information is not used in monaural hearing, but it is used in binaural, stereo hearing. Phases of the basilar membrane traveling wave play a crucial role in delays between left and right cochlea (Joris et al. 2006b; Vencovsky and Rund 2016). The cochlear delay theory is based on recordings from different nuclei of the auditory pathway including the spiral ganglion of the auditory nerve in cat.

Another alternative is *(3) broadly tuned channels* (Brand et al. 2002; Harper and McAlpine 2004). The theory states that few directions are combined in a population code, such as in three color vision channels in trichromats (Denman et al. 2017). The mechanism of broadly tuned channels in sound localization was proposed following experiments in the Mongolian gerbil *Meriones unguiculatus* by Brand et al. (2002). Apparently, different animals developed a multitude of mechanisms for sound localization.

Let us give one more example: the fly *(4) Ormia ochracea* has two eardrums less than 0.5 mm apart. Therefore, it resolves timing differences in the range of nanoseconds. This is accomplished by a lever mechanically comparing the two inputs (Mason et al. 2001).

*Vector strength* is an important parameter (Goldberg and Brown 1969). It can be found virtually at all levels of spike train description, which takes into account spike timing relative to the stimulus phase of another spike train. Marsalek (2001) discusses vector strength in the MSO nucleus at low frequencies. Vector strength can be described at the moment of mechanical-to-electrical transduction in cochlea (Camalet et al. 2000) and then at many places upwards in the auditory pathway and beyond.

*Convolution* is an important operator in signal processing. For the convolution calculation in the auditory pathway and comparison of beta and normal densities in this context, see Drapal and Marsalek (2010, 2011). The Laplace transform used in these two papers can be found in any standard calculus textbook, for example (Bronstein and Semendyaev 2013). The convolution of two spike time densities is used here for the ISI calculation. Another interpretation of the calculation and the parameters is possible - the output density also represents the probability of generating an output spike in a neural circuit of the MSO as a function of the time delay imposed into the circuit by the spike arrival difference from the neurons of the peripheral pathways from the left and right ears. The ensemble code produced this way signals the azimuth of the sound source localization.

The choice of the PDFs (normal, circular normal, beta, sine) in the calculations was made based on the correspondence between PDFs on the aperiodic versus on the periodic supports. The beta distribution was used because one of the authors and his colleague found an analytical result with the use of Laplace transform to describe spike computations based on the convolution of two beta distributions (Drapal and Marsalek 2010). Circular statistics is essential in the description of periodic data Kajikawa and Hackett (2005), Joris et al. (2006a), Sun et al. (2012) and Ahveninen et al. (2016).

It is generally assumed that neural coding uses either mean firing rate or spike timing. Mean firing rates were frequently attributed to labeled line encodings, sometimes also known as spatio-temporal codes (Koch and Laurent 1999). Timing codes were frequently demonstrated in a unitary situation, where one and only one spike encodes a complex visual information. This can be regarded as elementary or unitary encoding (Thorpe et al. 2001). Any timing code must survive time warping (Hopfield and Brody 2001; Nawrot et al. 2008). Brain pathways diverge and converge between several nuclei and it is also important, in which order the elementary computations are executed. Bures (2012) and Bures and Marsalek (2013) give comparison of these orderings between the LSO and the MSO nuclei.

### Ergodicity and neural signals

How can the ergodic property contribute and help this debate? *First* of all, experimental recording from the auditory brainstem is quite difficult. It is also difficult to record from several neurons instead of just one. Therefore, the population coding cannot be recorded directly. This situation calls for a construction of models. *Second*, the concept of the ideal observer can be used. The ideal observer has complete access to a signal relayed by a given population of neurons. This gives a precision that allows discrimination between input signals. The ideal observer is used in signal detection theory, in psychophysics and also in neural coding Tanner Jr. (1961), Green and Swets (1966), Geisler (2011) and Sanda and Marsalek (2012). *Third*, the ergodic assumption can be used in the comparison of experimental results in different species with different numbers of neurons involved in the nuclei of sound localization circuits. Even though the assumption of similar neural computation in animals with different head sizes is not obvious and it is already known that multitude of neural computations exist in sound localization pathways (Joris et al. 1998) ergodicity can bring unifying view at behavioral performances of different species.

*Next*, let us put this a bit more generally: When an input signal is processed in parallel, frequently it can be regarded as ergodic. Let us illustrate the opposite, which is serial processing, using an example outside neuroscience. We can compare encodings of a signal from multimedia processing by digital technology. We know that a digitally encoded signal takes a narrower radio transmission bandwidth, but also longer time to decode and that signal decoding requires a certain amount of computational power that has become available only recently, in comparison to analog transmission schemes (Haykin and Moher 2007; Meinel and Sack 2014). In this sense, digital encoding is intrinsic (implicit), which means the modality cannot be read out without computational decoding, which is likely not ergodic. This can be compared to the extrinsic (explicit) encoding of analog signals, which we expect to be ergodic. By this we want to point to possible cause, why neural codes are still poorly understood compared to genetic code. Frequently, the spike train modality serves as a *carrier* signal of the neurally transmitted intrinsic modality.

### Open questions for future research

Cochlear implants are successful in replicating a series of action potentials in the auditory nerve by imitation of the mechanical to electrical transduction in the cochlea by contemporary electronics (Drapal and Marsalek 2010). Constructing cochlear implants involves reverse biomimetic and neuromimetic engineering. The biomimetic and neuromimetic approaches are an engineering method to construct industrial sensors by mimicking nature’s solution to the problem. Both the reverse and neuromimetic engineering are very dependent on computational modeling, on the right choice of models and on their complexity. The knowledge that neural pathways calculate some quantity does not necessarily tell us how it is computed. Therefore, phenomenological models of the auditory brainstem computation are useful as a first approximation, which can be refined in subsequent research (Marsalek and Kofranek 2005).

Even though some formulas presented here as for example the formula for output PDF of neural circuit Eq. (21) are far from elegant, we urge our kind readers to direct their attention to the figures, which correspond to formulas and offer a more simplified view of the computations discussed here. Many other concepts from statistical physics besides ergodicity have been successfully employed in the description of neural computation, particularly in descriptions of sensory transduction into spike trains. We can look for ergodicity in many experiments dealing with time frequency/spatial frequency, and time/space separability, Lehky (1985). Another such example is Boltzmann’s proposal in 1876 to develop the equipartition principle. One of the authors with a colleague used the equipartition principle in descriptions of several disperse measures of spike timing in Kostal and Marsalek (2010). Neural codes are optimal, as has been demonstrated not only in the binaural hearing (Harper and McAlpine 2004; Ahveninen et al. 2014), but also in the olfactory system (Pokora and Lansky 2008). Information-theoretic approaches are infamous for the promiscuous and sometimes unnecessary use of complicated formulas. A somewhat more readable account on information-theoretic measures can be found in Kostal et al. (2011).

### Conclusions

We investigated the characteristics of the neuronal firing related to the sound phase in lower sound frequencies from 20 Hz to 2 kHz. We have solved a classic problem of the fitting of unknown parameters of a hypothetical neuronal firing distributions to experimental data. Starting from parameters of the beta density, we were able to describe the relation between the spike synchronization measured by the vector strength and the standard deviation of spike timing. Also, we described the relation between the interspike interval and the spike timing distributions.

## Abbreviations

CDF: Cumulative distribution function
CF: Characteristic frequency
IPD: Interaural phase difference
ISI: Inter-spike interval
ITD: Interaural time difference
LSO, MSO: Lateral and medial superior olive, twin nuclei in most mammalian brain stems
PDF: Probability density function

## List of symbols

$$a, b, c, d, A, B, C, K, N, \alpha , \beta , \delta $$: Parameters
$C_{\mathrm {V}}$: Coefficient of variation
$$\varphi $$: Sound phase
*f*: Frequency of periodic input, function
$$f_\mathrm {B}$$: Beta density
$$f_\mathrm {C}$$: Characteristic frequency
$$f_\mathrm {D}$$: Frequency difference
$f_\mathrm {S}$: Frequency of sound
$$f_{\sin }$$: Sine density
$f_{\max }$: Maximum sound frequency, here 10 kHz, human $f_{\max }$ is lower than 20 kHz
*f*, *g*, *h*: Functions
$\mathrm {H}$: Heaviside step function
*i*: Imaginary unit
$$I$$: Stimulus intensity
*R*: Firing rate
$$r$$: Vector strength
*s*: Scaling coefficient
$\sigma$: Standard deviation, when used in spike times, it is also called spike timing jitter
*t*: Time
*T*: Sound period
$$u$$: Weight coefficient
